# Silencing of *RUNX2* enhances gemcitabine sensitivity of *p53*-deficient human pancreatic cancer AsPC-1 cells through the stimulation of TAp63-mediated cell death

**DOI:** 10.1038/cddis.2015.242

**Published:** 2015-10-15

**Authors:** H Sugimoto, M Nakamura, H Yoda, K Hiraoka, K Shinohara, M Sang, K Fujiwara, O Shimozato, H Nagase, T Ozaki

**Affiliations:** 1 Laboratory of DNA Damage Signaling, Chiba Cancer Center Research Institute, 666-2 Nitona, Chuou-ku, Chiba 260-8717, Japan; 2 Laboratory of Cancer Genetics, Chiba Cancer Center Research Institute, 666-2 Nitona, Chuou-ku, Chiba 260-8717, Japan; 3 Innovative Therapy Research Group, Nihon University Research Institute of Medical Science, Nihon University School of Medicine, 30-1 Oyaguchi-Kamicho, Itabashi, Tokyo 173-8610, Japan

It has been well-known that human pancreatic cancer represents the fourth and fifth leading causes of cancer-related deaths in the United States and Japan, respectively.^1, 2^ Notably, pancreatic cancer is characterized by high metastatic potential, resistance to chemotherapy and thus its prognosis is extremely poor with 5-year survival <5%. At diagnosis, more than 80% cases are already advanced and non-resectable.^3^ Therefore, chemotherapy and/or radiotherapy is the only option. Despite improvements in the treatments, the survival rate has not been significantly ameliorated over the last few decades. For chemotherapy, a deoxycytidine analog termed gemcitabine (GEM) is the first line of standard treatment given to most of the patients bearing advanced pancreatic cancer.^4^ Unfortunately, GEM treatment provides limited clinical benefits, especially in advanced and metastatic disease.^5^ Hence, the extensive efforts to clarify the precise molecular mechanisms behind GEM-resistant phenotype of malignant pancreatic cancer and also to develop the promising strategies to enhance the efficacy of GEM should be required.

RUNX2 (Runt-related transcription factor 2) is one of the RUNX family members implicated in the induction of osteoblast differentiation and bone formation.^6^ Recently, we have found for the first time that RUNX2 attenuates p53/TAp73-dependent proper DNA damage response in *p53*-proficient human osteosarcoma-derived U2OS cells.^7, 8^ On the basis of our results, RUNX2 prohibited the transcriptional as well as pro-apoptotic activity of p53 through the complex formation with p53 in response to adriamycin (ADR). In addition, RUNX2 trans-repressed the transcription of *TAp73* following ADR exposure. Thus, our recent studies strongly suggest that RUNX2 has an oncogenic potential through the inhibition of DNA damage-dependent cell death pathway mediated by pro-apoptotic p53/TAp73. Consistent with the above-mentioned our notion, it has been described that RUNX2 has an ability to transactivate a subset of its target genes involved in cancer cell migration and invasion.^9^

In the current study, we have focused on human pancreatic cancer cells. According to our present observations, *p53*-deficient pancreatic cancer AsPC-1 cells exhibited a much more higher resistance to GEM as compared with *p53*-proficient pancreatic cancer SW1990 cells. Intriguingly, GEM treatment in AsPC-1 cells resulted in an induction and a reduction of pro-apoptotic TAp63 and pro-oncogenic RUNX2, respectively, indicating that there exists an inverse relationship between the expression levels of TAp63 and RUNX2 in response to GEM. Thus, it is likely that RUNX2 is capable to trans-repress *TAp63* transcription. Indeed, forced expression of RUNX2 in AsPC-1 cells markedly suppressed the transcription of *TAp63*. Of note, close inspection of 5′-upstream region of *TAp63* gene revealed that there exists a putative RUNX2-binding site (−553 to −548).^10^ In addition, forced expression of TAp63*α* in AsPC-1 cells significantly reduced the number of G418-resistant colonies as compared with control cells transfected with the empty plasmid. These observations raised a possibility that RUNX2-mediated repression of *TAp63* transcription might contribute to the acquisition and/or maintenance of GEM-resistant phenotype of ASPC-1 cells. To address this issue, siRNA-mediated knockdown of *TAp63* in AsPC-1 cells was performed. Our siRNA against *TAp63* efficiently reduced the expression of *TAp63* but not of transactivation-deficient Δ*Np63*. As expected, silencing of *TAp63* remarkably reduced the sensitivity of AsPC-1 cells to GEM relative to GEM-exposed non-silencing control cells. In support of these observations, depletion of *TAp63* attenuated GEM-dependent transactivation of a subset of p53/TAp63-target genes.

Considering that knockdown of *RUNX2* significantly enhances ADR sensitivity of U2OS cells,^7^ we have examined whether silencing of *RUNX2* could affect GEM sensitivity of AsPC-1 cells. On the basis of our present results, *RUNX2* knockdown enhanced GEM sensitivity of AsPC-1 cells accompanied by further accumulation of TAp63 as well as a subset of its target genes in response to GEM, implying that RUNX2-mediated trans-repression of *TAp63* has a pivotal role in the regulation of GEM-resistant phenotype of *p53*-deficient pancreatic cancer cells (Figure 1). It is worth noting that depletion of *TAp63* reduced GEM-mediated accumulation of DNA damage marker *γ*H2AX, whereas the amounts of *γ*H2AX was elevated in GEM-exposed *RUNX2* knockdown cells. As DNA damage-mediated phosphorylation of H2AX is mediated by phosphorylated ataxia telangiectasia mutated (ATM),^11^ we have checked the phosphorylation status of ATM in the presence or absence of GEM. Our immunoprecipitation/immunoblotting experiments clearly demonstrated that GEM-mediated phosphorylation of ATM is abrogated in *TAp63*-silencing cells, suggesting that TAp63 participates in the regulation of ATM-dependent phosphorylation of H2AX following GEM exposure. However, the precise molecular mechanisms how TAp63 contributes to ATM-dependent phosphorylation of H2AX in response to GEM remain elusive. Further studies should be required to adequately address this issue.

Taken together, our present findings strongly suggest that RUNX2 attenuates TAp63-dependent cell death pathway in *p53*-deficient pancreatic cancer cells following GEM exposure, and thus the depletion of *RUNX2* might be an attractive strategy to enhance the efficacy of the clinically approved GEM, which contributes to save cost to treat patients with advanced pancreatic cancer when compared with the development of novel anticancer drug(s) targeting pancreatic cancer.^12^

## Figures and Tables

**Figure 1:**
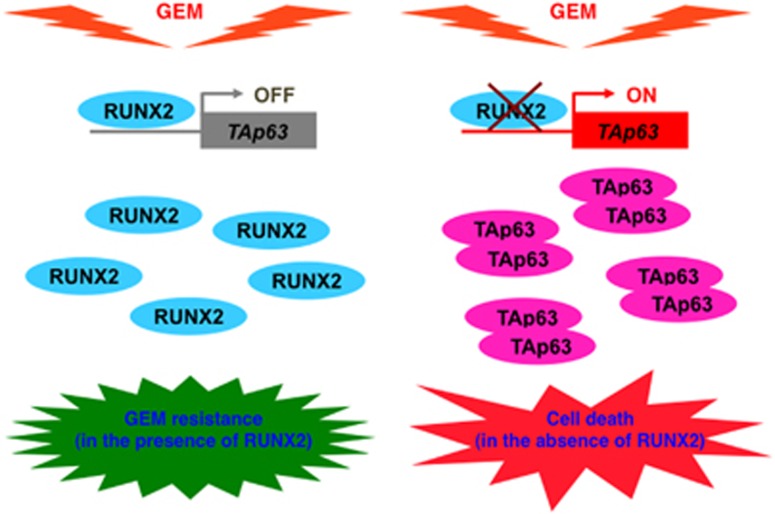
Depletion of *RUNX2* enhances gemcitabine sensitivity of *p53*-deficient human pancreatic cancer AsPC-1 cells through the stimulation of TAp63-mediated cell death pathway

## References

[bib1] 1Siegel R et al. CA Cancer J Clin 2014; 64: 9–29. 24399786 10.3322/caac.21208

[bib2] 2Kuroda T et al. BMC Gastroenterol 2013; 13: 134. 24256464 10.1186/1471-230X-13-134 PMC3766232

[bib3] 3Smeenk HG et al. Langenbecks Arch Surg 2005; 390: 94–103. 15578211 10.1007/s00423-004-0476-9

[bib4] 4Moss RA et al. Onco Targets Ther 2010; 3: 111–127. 20856847 10.2147/ott.s7203 PMC2939765

[bib5] 5Li Q et al. PLoS One 2014; 9: e104346. 25093849 10.1371/journal.pone.0104346 PMC4122434

[bib6] 6Komori T et al. Cell 1997; 89: 755–764. 9182763 10.1016/s0092-8674(00)80258-5

[bib7] 7Ozaki T et al. Cell Death Dis 2013; 4: e610. 23618908 10.1038/cddis.2013.127 PMC3641350

[bib8] 8Ozaki T et al. FEBS J 2015; 282: 114–128. 25331851 10.1111/febs.13108 PMC4368372

[bib9] 9Chimge NO et al. Oncogene 2013; 32: 2121–2130. 23045283 10.1038/onc.2012.328 PMC5770236

[bib10] 10Ito Y et al. Nat Rev Cancer 2015; 15: 81–95. 25592647 10.1038/nrc3877

[bib11] 11Falck J et al. Nature 2005; 434: 605–611.15758953

[bib12] 12Sugimoto H et al. Cell Death Discov 2015; 1: 15010. 10.1038/cddiscovery.2015.10 PMC4981025 27551445

